# Assessing Lead, Nickel, and Zinc Pollution in Topsoil from a Historic Shooting Range Rehabilitated into a Public Urban Park

**DOI:** 10.3390/ijerph14070698

**Published:** 2017-06-30

**Authors:** Ricardo Urrutia-Goyes, Ariadne Argyraki, Nancy Ornelas-Soto

**Affiliations:** 1 Departamento de Ciencias de la Energía y Mecánica, Universidad de las Fuerzas Armadas ESPE, Av. Gral. Rumiñahui s/n, P.O. Box 171-5-231B, Sangolqui 171103, Ecuador; 2 Laboratorio de Nanotecnología Ambiental, Centro del Agua para América Latina y el Caribe, Tecnológico de Monterrey, Monterrey 64849, Mexico; 3 Faculty of Geology and Geoenvironment, National and Kapodistrian University of Athens, Panepistimiopolis, Zographou, 157 84 Athens, Greece; argyraki@geol.uoa.gr

**Keywords:** health risk assessment, heavy metals, shooting range, soil contamination, trace elements

## Abstract

Soil contamination is a persistent problem in the world. The redevelopment of a site with a historical deposition of metals might conceal the threat of remaining pollution, especially when the site has become a public place. In this study, human health risk assessment is performed after defining the concentrations of Pb, Ni, and Zn in the topsoil of a former shooting range rehabilitated into a public park in the Municipality of Kesariani (Athens, Greece). A methodology that uses inductively coupled plasma mass spectrometry (ICP-MS, 13 samples), another that uses portable X-ray fluorescence (XRF) following a dense sample design (91 samples), and a hybrid approach that combines both, were used to obtain the concentrations of the trace elements. The enrichment factor and geoacummulation index were calculated to define the degree of pollution of the site. The hazard quotient and cancer risk indicators were also computed to find the risk to which the population is exposed. The present study reveals high non-carcinogenic health risk due to Pb pollution with ingestion as the main exposure pathway. The carcinogenic risk for Pb is within tolerable limits, but the definition of land use might alter such a statement. Lastly, regarding Ni and Zn, the site is unpolluted and there is insignificant carcinogenic and non-carcinogenic risks.

## 1. Introduction

The rapid development of the world has accelerated the evolution of urban areas and has also decreased the quality of the environment. Urban soil, specifically, serves as a home for many pollutants and, hence, is an indicator of increasing environmental contamination [1]. Soils from former shooting ranges have been reported to be enriched in some trace elements due to the metals present in ammunition released into the air and settled down to the ground. The potential contamination of a former shooting range can be overlooked when the site has already been redeveloped, but there is a chance that, even after many years, pollutants still remain in the site as a latent threat since trace elements can stay in the soil for decades and be subject to mixing processes, which can modify their bioavailability [2,3,4,5].

Heavy metals found in ammunition can have a direct influence on human health as they can be easily incorporated into the human body by ingestion, inhalation, or dermal absorption [6,7,8]. Elements including Pb, Zn and Ni are regularly studied in firing ranges since bullet cores contain Pb, and cartridges contain Zn and Ni [2,6,9,10]. The adverse effects on human health produced by these elements include renal, cardiovascular, and neurological complications due to the ingestion or inhalation of Pb; anemia and digestive diseases due to the ingestion of Zn; and Ni has been reported as a carcinogen [11,12,13]. Many studies concentrate on the characterization of heavy metals in soil focusing on element concentration and distribution [1,2,10,14,15,16,17,18,19,20,21,22,23,24,25,26,27,28,29,30,31]. Other studies focus on risk assessment [9,11,25,32,33,34,35,36,37,38,39] or firing ranges, specifically [2,3,10,17,37,38,39,40,41]. Detailed reports on human health risk assessment of trace elements in areas with a history of recurrent heavy metal accumulation are scarce. The presence of pollutants in rehabilitated areas with a background in contamination needs a continuous evaluation to define the health risks produced, in order to propose possible remediation actions or policies to preserve both environmental and human health.

A public park located in Kesariani (Athens, Greece) is an example of urban redevelopment. With decades of history, the site went from an execution site in the 1940s, to a military range in the 1970s, to an urban monument in the 2000s, and is nowadays a public park surrounded by a growing urban area. A portion of the site was redeveloped but some areas still remain intact. Many people gather around the park for leisure without any awareness of the environmental issues within the ground, as only few studies have been previously performed in the area with intriguing results [42,43]. The main goal of the present study is to perform a human health risk assessment (both carcinogenic and non-carcinogenic) after characterizing heavy metals (Pb, Ni, Zn) present in the topsoil of a shooting range that has undergone redevelopment into a public park in order to define the degree of hazard/risk to which the population is exposed. Taking into account the past of the site, other elements, such as Cu, As, Sb, and Cr could be of interest; however, preliminary analysis in the study area showed very low and homogeneous concentrations and, hence, were not included in this study.

## 2. Materials and Methods

### 2.1. Study Site Description

**Figure 1:**
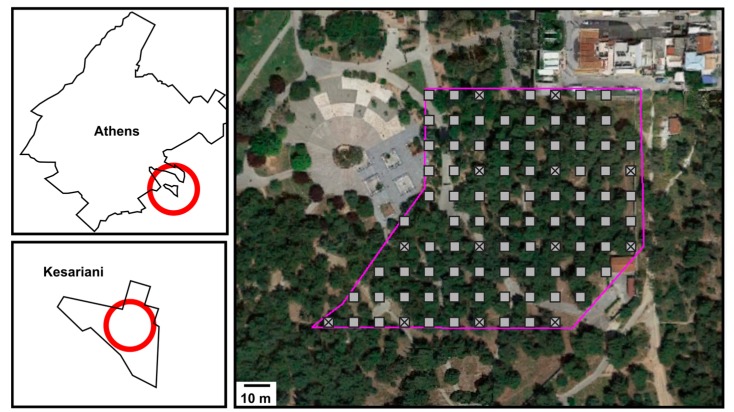
Study site in Athens. Ninety-one sampling locations for a methodology using X-ray fluorescence (XRF) are marked as gray squares, and 13 sampling locations for a methodology using ICP-MS are marked as crossed gray squares. The study area is outlined in purple, referenced on the field with a GPS and long measuring tape. Map data source: Google Maps.

The urban park is situated in the Municipality of Kesariani, a few kilometers east of the Athens center (see Figure 1). Its almost flat topography covers an area of ~0.7 km^2^ covered mostly by simple vegetation (i.e., grass and coniferous trees). Currently, the park is a historical monument, but in the past it was used as an execution site during World War II, subsequently served the military, and later became a recreational shooting range. After the redevelopment of the area in the 2000s, the park serves as a public place for recreation and many people visit it daily. The surrounding area has a well-urbanized landscape and it is a commercial bridge between the town and the Athens city center.

### 2.2. Sampling

Samples for this study were collected in 2016. The study area was sampled according to the following criteria (see Figure 1): (1) sample collection from 13 locations according to international regulations and measuring of elemental concentrations using ICP-MS (inductively coupled plasma mass spectrometry) following a total acid digestion; (2) sample collection from 91 locations (including the previous 13) in a denser design and measuring concentrations by using portable XRF; and (3) correct the 91 XRF measurements to match ICP-MS data values. Samples weighing ~400 g were removed from the area by digging through the topsoil (0–20 cm) using a spatula and removing any gravel and vegetation present. They were later stored in polypropylene bags. This sampling procedure corresponds to that recommended by international regulators in order to define metal concentrations in soils [44,45,46]. In the laboratory, samples were dried in a furnace at 60 °C for 24 h and disaggregated in a mortar. Sieving was carried out with a <2 mm plastic sieve (to avoid contamination) and quartering was performed to obtain smaller homogeneous subparts that were later sieved with a <250 µm mesh. Any loss of material during sieving was negligible.

### 2.3. Analytical Procedures

Three sets of data were obtained to compare their respective performances when assessing health risk at the study area: (a) 91 samples analyzed by portable XRF; (b) 13 samples analyzed by ICP-MS, and (c) 91 samples analyzed by the hybrid methodology.

#### 2.3.1. XRF Procedure

The concentration of 91 samples (see Figure 1) was measured by a portable X-ray fluorescence (XRF) device. Each sample was compacted inside a cylinder and placed inside a workstation to take the measurements for 90 s [47]. Different certified reference materials (CRM) measured 10 times with the device produced similar precision and accuracy estimates.

#### 2.3.2. ICP-MS Procedure

The metal concentration of 13 samples (see Figure 1) was measured using inductively coupled plasma mass spectrometry (ICP-MS) following guidelines by CCME [48] and USEPA [47]. Briefly, a pre-weighted amount of 0.2 g of fine-grained (<250 µm) soil was dissolved using hot (95 °C) aqua regia, followed by hydrofluoric and perchloric acid [27], and diluted into a 100 mL volumetric flask with deionized water. Quality control of the analytical results was performed using reagent blanks, CRMs, and duplicates.

#### 2.3.3. Hybrid Methodology

In order to define the hybrid methodology, the data of the 91 points obtained by using XRF were fitted to match the data of the 13 sampling points taken by using ICP-MS, according to Suh et al. [49]. Briefly, 13 sampling points were analyzed by both techniques, i.e., XRF and ICP-MS, and the correlation and linear regression were calculated between them. Subsequently, the sampling points for XRF were extended to 78 more (i.e., 91 in total). Thus, the trend equations (*y* = 0.2658*x* for Pb, *y* = 1.3452*x* for Ni, and *y* = 1.1674*x* for Zn; *R*^2^ > 0.90 in every case; where *y* is XRF data and *x* is ICP-MS data) were used to transform the rest of the XRF data values into corresponding ICP-MS data values.

### 2.4. Reagents and Instrumentation

All experiments were performed using reagent-grade chemicals and deionized water. Hydrochloric acid, nitric acid, and standard stock solutions used were obtained from Sigma-Aldrich (St. Louis, MO, USA). CRMs were obtained from NIST^®^ (Gaithersburg, MD, USA) and AccuStandards^®^ (New Haven, CT, USA). Instrumental analysis was carried out by ICP-MS using a Thermo Fisher (Waltham, MA, USA) X Series 2 instrument while XRF analysis was performed using an Olympus (Newton, MA, USA) Delta Premium 6000 device.

### 2.5. Pollution Indicators

Enrichment factor (*EF*) and Geoacummulation index (*I_geo_*) were used to evaluate the degree of pollution at the site [32,50,51]. *EF* is calculated according to Equation (1), where *n* and *ref* denote the target metal and its reference value, respectively, defined both in the sample and in the background. *EF* values suggest anthropogenic sources when its value is higher than 10. Table 1 shows the different categories recognized according to the *EF* value calculated [52,53]. The reference element used for normalization was Mn since it presented low variability. On the other hand, *I_geo_* is calculated according to Equation (2), where *C_n_* and *B_n_* are the concentrations of the target metal in the site and the background respectively. Table 1 shows the different classification of soil pollution according to the *I_geo_* value [32,51]. Concentrations from background material might be obtained from Rudnick and Gao [54], or other sources, if available. In the present study, reference values were taken from a recent work held in Athens, Greece [18], which included park and woodland areas.

(1) $$EF=\frac{{\left(C_{n}/C_{ref}\right)}_{sample}}{{\left(B_{n}/B_{ref}\right)}_{background}}$$

(2) $$I_{geo}=log_{2}\left(\frac{C_{n}}{1.5\times B_{n}}\right)$$

### 2.6. Human Health Risk Assessment

Assessing the health risk regarding the studied metals requires the definition of population exposure in the study area. In a situation of recreation, exposure can occur via direct ingestion, inhalation, or dermal absorption of the elements when soil particles come in contact with the mouth, nose, or skin. However, the exposure frequency for recreation is lower than that of regular residential use. Indicators of health risk used in this study were those based on the work by USEPA [8,55]. The hazard quotient (*HQ*) and cancer risk (*CR*) for metals with non-carcinogenic and carcinogenic effects, were calculated, respectively, based on their corresponding chronic daily intake (*CDI*), reference dose (*RfD*), and slope factor (*SF*) values. Since the adverse effects produced by each element can be considered accumulative, indicators for each exposure pathway were summed [32]. The hazard posed by an element is considered low when its summed *HQs* (for ingestion, inhalation, and dermal contact) total less than one while, on the other hand, carcinogenic risk is considered tolerable when the calculated *CR* is in the range of 10^−6^–10^−4^ [11,32,33,34,55].

*CDI* values (the amount of chemical substance received by a person over a specific period of time) for ingestion (*CDI_ing_*), inhalation (*CDI_inh_*), and dermal contact (*CDI_dermal_*) were calculated following Equations (3)–(5), where the parameters used can be seen in Table 2. Finally, *HQ* and *CR* were calculated according to Equations (6) and (7), where *RfD* refers to the maximum daily exposure that would not create noticeable effects to the human population, and *SF* yields the incremental chance of a person to develop cancer over a lifetime under the described exposure [8,11,32,33,34,55]. *RfD* and *SF* are element- and ingestion pathway-dependent and can be seen in Table 3.

(3) $$CDI_{ing}=C_{exp}\times \frac{R_{ing}\times EF\times ED}{BW\times AT}\times 10^{-6}$$

(4) $$CDI_{inh}=C_{exp}\times \frac{R_{inh}\times EF\times ED}{PEF\times BW\times AT}$$

(5) $$CDI_{dermal}=C_{exp}\times \frac{SA\times SAF\times ABS\times EF\times ED}{BW\times AT}\times 10^{-6}$$

(6) HQ=CDIRfD

(7) CR=CDI×SF

### 2.7. Statistical and Spatial Analysis

Measured concentrations of all the elements followed a log-normal distribution. Descriptive statistics were calculated for the three studied elements including median, mean, and standard deviation. The software package used was Minitab^®^ (State College, PA, USA). Spatial representation for the human health risk assessment was performed using the SADA software of The University of Tennessee Knoxville (Knoxville, TN, USA).

## 3. Results and Discussion

Three different approaches were used to define concentrations for Pb, Ni, and Zn in the study area. Using either (1) a portable XRF technique on a dense sampling design; (2) an ICP-MS technique on a sampling design suggested by regulators, or (3) a hybrid approach complementing both techniques. The differences when performing human health risk assessments with the three approaches is discussed below.

### 3.1. Heavy Metal Concentrations

Regarding portable XRF performance, RSD values ranged from 1% to 2% for Pb, from 1% to 6 % for Zn, and from 8% to 18% for Ni; and mean recoveries calculated were 108%, 106%, and 135% for Pb, Zn, and Ni, respectively. Concerning ICP-MS, accuracy and precision analysis showed RPD values from 5% to 8% and mean recoveries from 98% to 102%.

**Figure 2:**
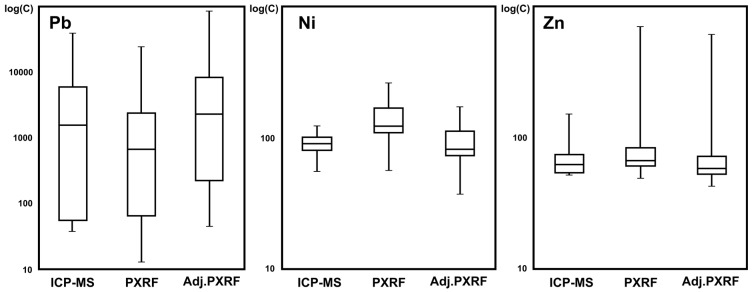
Box and whiskers plots of concentrations (in mg/kg) of the studied metals in surface soil of Kesariani Park, Greece.

Boxplots regarding element concentrations in the study area are presented in Figure 2. It can be noted that the mean concentration of Pb is 5560, 2043, and 7160 mg/kg when using the methodologies based on ICP-MS, XRF, or hybrid, respectively. The element concentrations in general, followed the order Pb > Ni > Zn. On the other hand, SD values for Pb are greater than the mean values, indicating that the data are spread out and that there is a high degree of heterogeneity in the site [56,57], whereas SD values for Ni and Zn are smaller than the mean values, suggesting a more homogenous distribution across the study area. The hybrid data and analysis for characterization can be considered the most convenient approach as it complements the accuracy of ICP-MS measurements with the numerous readings taken by XRF.

### 3.2. Pollution Indicators

**Figure 3:**
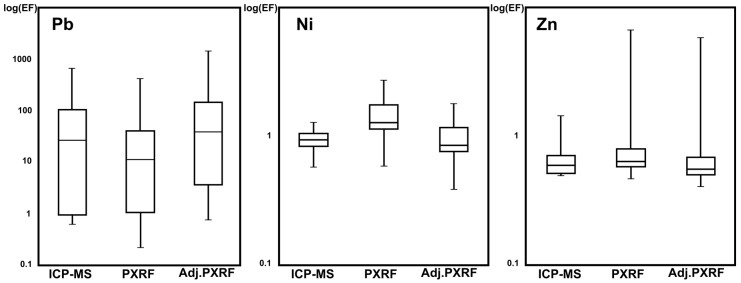
Enrichment factor values (*EF*) for the studied metals in Kesariani, Greece.

Enrichment factor and geoaccumulation index values can be seen in Table 4. Using mean values [32], *EF* values found for Ni and Zn by using any of the three aforementioned approaches, show minimal enrichment and, thus, suggest insignificant anthropogenic influence. While in the case of Pb, extremely high enrichment was found using both ICP-MS and hybrid-based methodologies. By contrast, very high enrichment was found by using the XRF-based methodology. However, taking into account the minimum and maximum *EF* values (see Table 4) it can be seen that, in some spots of the study area, the enrichment for Ni and Zn can be classified as moderate and significant, respectively, while, in others, the enrichment for Pb shows minimal impact. Nonetheless, as can be confirmed in Figure 3, in general, the majority of the *EF* values for Ni and Zn are smaller than two and *EF* values for Pb are mostly greater than two. Advantages of using synergistic procedures can be shown in this work, since the enrichment in the site is even higher that those predicted by the two basic methodologies independently. In general, *EF* values for Pb confirm the anthropogenic effects of the historical metal deposition activities held in most of the study area.

Additionally, all data concur on Pb pollution of the site based on the geoaccumulation index. In the case of ICP-MS and hybrid approaches, results allow the conclusion of extreme pollution while the XRF-based methodology denotes heavy to extreme pollution. One more time, a hybrid approach yields a more realistic definition of the pollution. Lastly, even though some locations show positive *Igeo* values for Ni and Zn, the site can be classified as unpolluted using mean values, regardless of the methodology used.

### 3.3. Health Risk Assessment

Table 5 shows carcinogenic and non-carcinogenic health risks calculated for adults and children, using the three methodologies abovementioned. In the case of *HQ* values, the corresponding contributions for each exposure pathway are also included. In all cases, it can be noted that the contribution from ingestion is higher than dermal contact and inhalation; and that children face higher non-carcinogenic threats compared to adults.

Ni and Zn show a low non-carcinogenic risk for the study area as their corresponding total *HQ* values are lower than one for both adults and children. This tendency is preserved regardless of the methodology being used. On the contrary, the non-carcinogenic risk calculated for Pb is mostly high as total *HQ* values are greater than those for adults (range from 1.15 to 2.92) and children (range from 3.04 to 27.1) regardless of the methodology with one exception: the calculated risk for adults using the XRF-based methodology, shows a total *HQ* of 0.33 and invites further discussion (see Section 3.4). It has been shown here that the three methodologies for characterization suggest similar conclusions regarding human health risks although to different degrees. However, since the hybrid methodology can be considered the most suitable for characterization, its use for health risk assessment should be the most effective. It can be said then, that the methodology based on ICP-MS overestimates the risks and the other based on XRF underestimates the risks.

With respect to carcinogenic elements, regardless of the methodology used, *CR* values for Ni are within an order of magnitude (10^−10^–10^−9^) for both age groups (see Table 5), suggesting insignificant risk. Contrastingly, *CR* values for Pb (10^−6^–10^−5^) fall within the tolerable criteria for both adults and children. This way, it has been established that Pb pollution with high non-carcinogenic risk for human health is present in the study area due to former recurrent metal deposition activities even though the area was redeveloped.

These results can be compared with others published in different cities around the world, even though the exposure frequency value used differs [29,32,33,34,58] (see Table 6). For instance, *HQ* values for Pb are generally higher by a factor of ten both for adults and children from Zhuzhou, China; *HQ* values calculated for Ni are higher than the ones reported in Madrid, Spain; and *HQ* values for Zn are in the same order of magnitude than those reported in Madrid, Spain. The elevated health risk due to the presence of Pb in Kesariani can be readily seen when comparing the indicators with those reported in Thessaloniki, a Greek city located to the north of the country, since *HQ* values are much higher in the study area. Nonetheless, health risk indicators for Ni and Zn are greater in Thessaloniki.

More detailed studies regarding playgrounds/parks or shooting ranges are scarce and unspecific. This way, five studies performed on playgrounds of different cities can also be seen in Table 6. Although *EF* values used for calculations differ (since recreational habits vary greatly between regions), it can be noted that *HQ* and *CR* values calculated for the study area are greater in every case. On the other hand, three studies performed on shooting ranges can also be seen in Table 6. In this regard, it can be noted that the results from this work are congruent with those reported in Canada and Finland since *HQ* values for Pb range around the unity. These results suggest that the human health risk indicators calculated for Pb are definitely affected by the previous activity taken place in the study area and the importance of monitoring public areas with a history of metals deposition.

### 3.4. Land Use and Its Effects on Human Health Risk Assessment

It was mentioned in Section 3.3 that some calculated *HQ* values are close to the suggested threshold levels. In this regard, a short analysis on the influence of the assigned land use on health risk assessment seems convenient. Section 2.6 described the parameters set for *CDI* calculations. The exposure frequency (*EF*) was defined as 40 days/year as the study area can be considered for recreational use. However, as the neighboring area of the park is densely populated, the parameter *EF* can be set up to 180 days/year for residential use (see Supplementary Materials, Table S1). In such case, *HQ* values could increase and surpass the threshold reference value, e.g., the total *HQ* for adults using the XRF-based approach could increase from 0.33 (low risk) to 1.48 (high risk) based on the determination of land use of the study area. Similarly, *CR* values for children and adults obtained with the application of the methodology based on ICP-MS, and *CR* values for children found using the hybrid approach are 6.78 × 10^−5^, 2.90 × 10^−5^, and 2.67 × 10^−5^, respectively. These values could also be increased by defining the study area as residential and resulting *CR* values would be 1.20 × 10^−4^, 1.31 × 10^−4^, and 3.05 × 10^−4^, which fall out of the tolerable window for safety. It is important to be aware of this influence as the designation of land use could be subjective.

### 3.5. Spatial Representation of Risk for Pb

**Figure 4:**
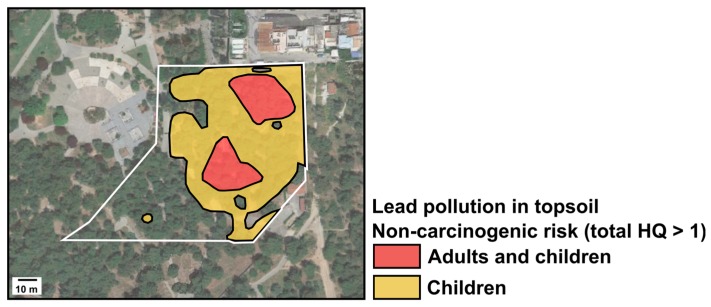
Spatial representation of non-carcinogenic risk due to Pb contamination based on total *HQ* values for adults and children in Kesariani, Greece. The study area is delineated in white. Map data source: Google Maps.

The main threat found in the site corresponds to non-carcinogenic risk due to the presence of Pb. In this regard, since the hybrid methodology for characterization has been defined as the most convenient, a spatial representation of such risk can be performed to show areas where the risk is higher and where precautions should be taken. Even though the study site is a small public area, the exercise here described can serve as an example on how to define the spatial risk assessment at larger scales. Figure 4 shows the spatial representation of non-carcinogenic risk due to Pb contamination based on total *HQ* values for adults and children.

## 4. Conclusions

The present study has exposed high non-carcinogenic health risk due to Pb contamination in the studied part of Kesariani Park (Athens, Greece). Three methodologies were implemented in order to contrast their performance and the site was, in the first instance, considered a recreational area. The calculated risk indicators are based on trace elements found in topsoil, which belongs to an area with historical heavy metal deposition activities. Although both adults and children are exposed to such non-carcinogenic risk, the threat for children is more widespread, spatially, and shows a hazard quotient value almost ten times higher. *HQ* values show that the main exposure pathway of concern is ingestion, followed by dermal contact and inhalation regardless of the age group. On the other hand, *CR* values indicate a cancer risk within tolerable levels, but deeper analysis has led to the suggestion that land use is an important factor when performing health risk assessments. Lastly, the site has also been defined as unpolluted for Ni and Zn. *HQ* and *CR* values have led to the conclusion that there is negligible carcinogenic and non-carcinogenic risks regarding both metals.

## Figures and Tables

**Table 1:** Classification of pollution indicators.

Category of Enrichment	*EF*	Category of Pollution	*I_geo_*
Minimal	<2	Unpolluted	<0
Moderate	2–5	Unpolluted to moderate	0–1
Significant	5–20	Moderate	1–2
Very high	20–40	Moderate to heavy	2–3
Extremely high	>40	Heavy	3–4
		Heavy to extreme	4–5
		Extreme	>5

*EF*: Exposure frequency, *I_geo_*: Geoacummulation index.

**Table 2:** Description of parameters and values used [8,11,31,32,33,54] in human health risk assessment in Kesariani, Athens.

Parameter	Name	Units	Value
***C_exp_***	Concentration of the trace element	mg/kg	Element dependent
***R_ing_***	Ingestion rate	mg/day	200 for children, 100 for adults
***EF***	Exposure frequency	days/year	40 for recreational
***ED***	Exposure duration	years	6 for children, 24 for adults
***BW***	Body weight	kg	15 for children, 70 for adults
***AT***	Averaging time	days	*ED* × 365
***R_inh_***	Inhalation rate	m^3^/day	7.5 for children, 20 for adults
***PEF***	Particle emission factor	m^3^/kg	1.36 × 10^9^
***SA***	Exposed skin area	cm^2^/day	2800 for children, 5700 for adults
***SAF***	Skin adherence factor	mg/cm^2^	0.2 for children, 0.07 for adults
***ABS***	Dermal absorption factor		0.001 for non-carcinogenic, 0.01 for carcinogenic

**Table 3:** Parameters for *HQ(*hazard quotient*)* and *CR(*cancer risk*)* calculations [9,32].

Parameters (mg/kg/day)		Pb	Zn	Ni
***RfD***	Ingestion	3.50 × 10^−3^	3.00 × 10^−1^	2.00 × 10^−2^
	Inhalation			9.00 × 10^−5^
	Dermal contact	5.25 × 10^−4^	6.00 × 10^−2^	5.40 × 10^−3^
***SF***		8.50 × 10^−3^		8.40 × 10^−1^

*RfD*: reference dose; *SF*: slope factor.

**Table 4:** Pollution indicators for the studied elements in Kesariani, Greece.

Element	Reference Concentration	*EF*				*I_geo_*			
	(mg/kg) [18]	Min	Max	Mean	Category	Min	Max	Mean	Category
ICP-MS (*n* = 13)									
Pb	60	0.59	639	89	extr. high	−1.30	8.78	5.95	extreme poll
Ni	95	0.56	1.26	0.92	minimal	−1.36	−0.20	−0.65	unpolluted
Zn	104	0.48	1.41	0.65	minimal	−1.58	−0.04	−1.15	unpolluted
XRF (*n* = 91)									
Pb	60	0.20	400	32	very high	−2.85	8.11	4.50	heavy/extreme
Ni	95	0.57	2.66	1.42	minimal	−1.34	0.87	−0.03	unpolluted
Zn	104	0.46	6.66	0.80	minimal	−1.67	2.20	−0.85	unpolluted
HYBRID (*n* = 91)									
Pb	60	0.71	1402	115	extr. high	−1.04	9.92	6.31	extreme poll
Ni	95	0.38	1.76	0.94	minimal	−1.94	0.28	−0.63	unpolluted
Zn	104	0.39	5.77	0.70	minimal	−1.88	1.99	−1.06	unpolluted

Categories based on mean values.

**Table 5:** Human health risk assessment indicators (*HQ* and *CR*) for the studied elements in Kesariani, Greece.

Element	C 95%UCL	Adults					Children				
		*HQ*				*CR*	*HQ*				*CR*
		Ing	Inh	Derm	Total (∑)		Ing	Inh	Derm	Total (∑)	
ICP											
Pb	63,650.80	2.85	8.37 × 10^−4^	7.57 × 10^−2^	2.92	2.90 × 10^−5^	2.66 × 10^−1^	1.48 × 10^−3^	4.96 × 10^−1^	27.1	6.78 × 10^−5^
Ni	102.44	8.02 × 10^−4^	2.36 × 10^−7^	1.19 × 10^−5^	8.14 × 10^−4^	1.36 × 10^−9^	7.48 × 10^−3^	4.18 × 10^−7^	7.76 × 10^−5^	7.56 × 10^−3^	6.02 × 10^−10^
Zn	82.25	4.29 × 10^−5^	1.26 × 10^−8^	8.56 × 10^−7^	4.38 × 10^−5^		4.01 × 10^−4^	2.24 × 10^−8^	5.61 × 10^−6^	4.06 × 10^−4^	
XRF											
Pb	7,152.74	0.32	9.41 × 10^−5^	8.51 × 10^−3^	33.0 × 10^−2^	3.26 × 10^-6^	2.99	1.67 × 10^−4^	5.57 × 10^−2^	3.04	7.61 × 10^−6^
Ni	146.95	1.15 × 10^−3^	3.38 × 10^−7^	1.70 × 10^−5^	1.17 × 10^−3^	1.95 × 10^-9^	1.07 × 10^−2^	6.00 × 10^−7^	1.11 × 10^−4^	1.08 × 10^−2^	8.64 × 10^−10^
Zn	89.34	4.66 ×10^−5^	1.37 × 10^−8^	9.30 × 10^−7^	4.76 × 10^−5^		4.35 × 10^−4^	2.43 × 10^−8^	6.09 × 10^−6^	4.41 × 10^−4^	
HYBRID											
Pb	25,067.49	1.12	3.30 × 10^−4^	2.98 × 10^−2^	1.15	1.14 × 10^−5^	10.5	5.85 × 10^−4^	1.95 × 10^−1^	10.7	2.67 × 10^−5^
Ni	97.28	7.61 ×10^−4^	2.24 × 10^−7^	1.13 × 10^−5^	7.73 × 10^−4^	1.29 × 10^−9^	7.11 × 10^−3^	3.97 × 10^−7^	7.37 × 10^−5^	7.18 × 10^−3^	5.72 × 10^−10^
Zn	77.32	4.03 × 10^−5^	1.19 × 10^−8^	8.05 × 10^−7^	4.12 × 10^−5^		3.77 × 10^−4^	2.10 × 10^−8^	5.27 × 10^−6^	3.82 × 10^−4^	

C 95%UCL: 95% upper confidence level, *HQ*: hazard quotient, *CR*: cancer risk, Ing: ingestion; Inh: inhalation; Derm: dermal contact. Indicators above tolerable levels in bold.

**Table 6:** Comparison of human health risk assessment indicators (*HQ* and *CR*) in different cities for the studied metals.

City	Type of	Exposure	Pb				Ni				Zn	
	Study Area	Frequency (*EF*)	*HQ*		*CR*		*HQ*		*CR*		*HQ*	
		Used in Days/Year	Adults	Children	Adults	Children	Adults	Children	Adults	Children	Adults	Children
Kesariani, Greece	Former shooting	40	1.15	10.7	1.14 × 10^−^^5^	2.67 × 10^−^^5^	7.73 × 10^−^^4^	7.18 × 10^−^^3^	1.29 × 10^−^^9^	5.72 × 10^−^^10^	4.12 × 10^−^^5^	3.82 × 10^−^^4^
(this study)	range/park	180 *	5.18	48.0	5.15 × 10^−^^5^	1.20 × 10^−^^4^	3.48 × 10^−^^3^	3.23 × 10^−^^2^	5.81 × 10^−^^9^	2.57 × 10^−^^9^	1.85 × 10^−^^4^	1.72 × 10^−^^3^
Thessaloniki, Greece [32]	Urban street dust from commercial city	180	6.12 × 10^−^^2^	4.60 × 10^−^^1^			4.35 × 10^−^^3^	3.58 × 10^−^^2^	6.43 × 10^−^^9^	2.85 × 10^−^^9^	1.54 × 10^−^^2^	1.21 × 10^−^^2^
Zhuzhou, China [58]	Urban street dust from industrial city	180	4.36 × 10^−^^1^	3.17			1.99 × 10^−^^3^	1.45 × 10^−^^2^	4.90 × 10^−^^9^	6.76 × 10^−^^9^	1.12 × 10^−^^2^	8.09 × 10^−^^2^
Luanda, Angola [33]	Urban street dust from industrial city	180		7.23 × 10^−^^1^				3.55 × 10^−^^3^		4.61 × 10^−^^10^		7.40 × 10^−^^3^
Madrid, Spain [34]	Surface soil from playgrounds/park	27		3.11 × 10^−^^2^				9.61 × 10^−^^4^		1.37 × 10^−^^9^		7.10× 10^−^^4^
Istanbul, Turkey [59]	Surface soil from playgrounds/park	50–180	<1.00	<1.00			<1.00	<1.00			<5.0 × 10^−^^3^	<1.0 × 10^−^^3^
Lisbon, Portugal [60]	Surface soil from playgrounds	19–33		1.80								
Podgorica, Montenegro [61]	Surface soil from playgrounds	360		2.50 × 10^−^^1^								
Xiamen, China [11]	Surface soil from urban parks	75		1.23 × 10^−^^1^	4.66 × 10^−^^7^			5.00× 10^−^^3^	6.45 × 10^−^^10^			4.00 × 10^−^^3^
Ontario, Canada [62]	Firing range	nr	1.84–4.10									
Finland [37]	Firing range	30–90	0.90–1.20									
New York, USA [39]	Firing range	nr	<1.00		1.00 × 10^−^^11^–2.00 × 10^−^^5^						<1.00	

* See Table S1 in Supplementary Materials. nr: Not reported.

## Supplementary Materials

The following are available online at www.mdpi.com/1660-4601/14/7/698/s1, Table S1: Human health risk assessment indicators (*HQ* and *CR*) in Kesariani, Greece considering an exposure frequency (*EF*) of 180 days/year.
