# Supplementary material for: Assessing Lead, Nickel, and Zinc Pollution in Topsoil from a Historic Shooting Range Rehabilitated into a Public Urban Park
Source: Int J Environ Res Public Health. 2017 Jun 30;14(7):698. doi: 10.3390/ijerph14070698 (PMC5551136; doi:10.3390/ijerph14070698)
Supplement: Supplementary file 1 [file ijerph-14-00698-s001.pdf]

Supplementary Material

Table 1S. Human health risk assessment indicators (HQ and CR) in Kesariani, Greece considering an exposure frequency (EF) of 180 days/year.

Element	C 95%UCL	Adults					Children				
		HQ				CR	HQ				CR
		ing	inh	derm	total (∑)		ing	inh	derm	total (∑)	
ICP											
Pb	63,650.80	1.28E+01	3.77E-03	3.41E-01	**1.32E+01**	**1.31E-04**	1.20E+02	6.68E-03	2.23E+00	**1.22E+02**	**3.05E-04**
Ni	102.44	3.61E-03	1.06E-06	5.33E-05	3.66E-03	6.11E-09	3.37E-02	1.88E-06	3.49E-04	3.40E-02	2.71E-09
Zn	82.25	1.93E-04	5.68E-08	3.85E-06	1.97E-04		1.80E-03	1.01E-07	2.52E-05	1.83E-03	
XRF											
Pb	7152.74	1.44E+00	4.23E-04	3.83E-02	**1.48E+00**	1.47E-05	1.34E+01	7.51E-04	2.51E-01	**1.37E+01**	3.43E-05
Ni	146.95	5.18E-03	1.52E-06	7.65E-05	5.25E-03	8.77E-09	4.83E-02	2.70E-06	5.01E-04	4.88E-02	3.89E-09
Zn	89.34	2.10E-04	6.17E-08	4.19E-06	2.14E-04		1.96E-03	1.09E-07	2.74E-05	1.99E-03	
HYBRID											
Pb	25,067.49	5.05E+00	1.48E-03	1.34E-01	**5.18E+00**	5.15E-05	4.71E+01	2.63E-03	8.79E-01	**4.80E+01**	**1.20E-04**
Ni	97.28	3.43E-03	1.01E-06	5.06E-05	3.48E-03	5.81E-09	3.20E-02	1.79E-06	3.32E-04	3.23E-02	2.57E-09
Zn	77.32	1.82E-04	5.34E-08	3.62E-06	1.85E-04		1.69E-03	9.47E-08	2.37E-05	1.72E-03	

HQ: hazard quotient, CR: cancer risk. Indicators above tolerable levels are in bold.
